# Cost-effectiveness of population-wide screening for intracranial aneurysms revisited in light of potential diagnostic developments

**DOI:** 10.1177/17474930251344506

**Published:** 2025-05-12

**Authors:** Michael Veldeman, Oliver Schoeffski, Anke Hoellig, Gabriel J E Rinkel

**Affiliations:** 1 Department of Neurosurgery, RWTH Aachen University Hospital, Aachen, Germany; 2 School of Business and Economics, Chair of Health Care Management, Friedrich-Alexander University of Erlangen-Nuremberg, Nuremberg, Germany; 3 Department of Neurosurgery, University Hospital Mannheim, Medical Faculty Mannheim, University of Heidelberg, Mannheim, Germany; 4 Department of Neurology, UMC Utrecht Brain Center, University Medical Center Utrecht, Utrecht University, Utrecht, The Netherlands

**Keywords:** Intracranial aneurysm, subarachnoid hemorrhage, population-wide screening, magnetic resonance angiography, computed tomography angiography, quality-adjusted life years, willingness-to-pay

## Abstract

**Background:**

Preventive treatment of unruptured intracranial aneurysms (UIAs) has the potential to reduce aneurysmal subarachnoid hemorrhage (SAH) incidence. Population-wide screening (PWS) for UIAs has been disregarded, as it remains unclear how to manage low-risk UIAs. Higher cost for SAH treatment, along with improvements in UIA treatment decision-making, might improve the risk–benefit and cost–benefit ratios for PWS. Currently, blood-based screening tests for UIAs are under development and might be suitable for use in PWS.

**Aims:**

This study sets out to identify what health economic criteria should be met by a hypothetical UIA screening test to justify PWS.

**Methods:**

A Markov model was built to compare PWS versus standard of care. Model parameterization was done using real-world data derived from the population cared for by the RWTH Aachen University Hospital. Data in relation to SAH were derived from a prospective registry of consecutive SAH patients (n = 275). In addition, a database of newly diagnosed UIAs was retrospectively collected (n = 139). Incremental cost-effectiveness ratios (ICERs) were calculated to illustrate the annual cost per additional quality-adjusted life year (QALY). Sensitivity analyses were performed to determine at which price point the PWS strategy would become cost-effective based on different levels of willingness-to-pay (WTP).

**Results:**

In a one-way sensitivity analysis, the price of a hypothetical screening test was varied between €1 and €811.3 (mean cost of magnetic resonance angiography). In case of a WTP of €50,000 per QALY gained, the cost per test may be €225.72 and remain cost-effective. If the same test could also be used for watchful-waiting in low-risk patients (i.e. assess the risk of aneurysm growth), the price may increase up to €294.19. There is no price point at which PWS would become dominant and yield negative ICERs.

**Conclusion:**

PWS for UIAs is unlikely to be cost-effective, even with new blood screening technologies. However, once patents expire, and price monopolies are broken, use of such technologies may become more attractive for health policymakers, depending on their WTP.

## Introduction

Subarachnoid hemorrhage (SAH) caused by rupture of an intracranial aneurysm, is a major cause of cerebrovascular-related morbidity and death.^1^ Preventive treatment of unruptured intracranial aneurysms (UIAs) has the theoretical potential to eliminate SAH.^2^ Development of non-invasive angiographic imaging enables screening for UIAs without the risks associated with conventional cerebral digital subtraction angiography (DSA).

The economic implication of aneurysm screening using conventional methods has been assessed for populations a priori at higher risk of carrying UIAs.^3,4^ Based on economic evaluation, ethical considerations, and expert consensus, screening for UIAs is only recommended in patients with two or more affected first-degree relatives.^5,6^

Currently, blood-based tests, to detect the presence of cerebral aneurysms, are under development.^7^ In a hypothetical cost-effectiveness analysis (CEA), a cost of $34,515.13 per additional quality-adjusted life year (QALY) was calculated, if a (e.g. blood based) test assessing rupture risk, was used to follow up on low-risk UIA patients, and priced at $3951.^8^ This study did not account for the need and cost of further diagnostic exploration in case of a positive test result. Also, in most previous CEAs, costs and probabilities were extracted from different literature reports leading to input data originating from various populations which can obscure important population-specific trends.^3,4,8^ Moreover, estimated costs of SAH are oftentimes based on aneurysm treatment alone, overlooking considerable costs of intensive care treatment, neurorehabilitation and long-term care of the disabled.

UIAs are typically identified incidentally, when cranial imaging is performed for secondary reasons such as headache, vertigo, or minor head trauma. Only a minority of aneurysms will cause symptoms other than rupture.^9^ Population-wide screening (PWS) is expected to identify high volumes of asymptomatic UIA carriers of which the majority of aneurysms will not cause SAH. Once diagnosed, it remains unclear whether to prophylactically treat or not and patients with assessed low rupture risk are typically followed with sequential imaging. Here, amending costs are generated from follow-up over long periods of time. However, in recent years, rupture risk assessment has become more refined and patient-tailored.^10,11^ In addition, intensive care unit (ICU) treatment has become more expensive, which in turn could make a strategy favoring screening and prophylactic treatment, more cost-effective.^12^

### Objectives

This study sets out to identify what health economic criteria should be met by a hypothetical UIA screening test to justify PWS.

## Methods

### Study population

A Markov model was built to compare PWS versus standard-of-care (SOC) strategies, from a health care payer’s perspective. For model parameterization, real-world data were derived from the population cared for by the RWTH Aachen University Hospital (UKA). The UKA is a tertiary referral hospital and the sole provider of SAH care in its region. The hospital’s geographical drainage area is fairly fixed partly by its international borders to the east and is estimated as of 2022, at approximately 1,095,225 inhabitants (Supplemental Table 1). Costs, probabilities, and outcome data were extracted from two UKA patient cohorts. Data in relation to aneurysm rupture were derived from a prospective registry of consecutive patients with aneurysmal SAH, collected over 6 years (AC-SAH; 2014–2020; n = 275). Health outcome 1 year after aneurysm rupture was recorded using the modified Rankin scale (mRS) and converted into health utilities between 0 and 1.^13,14^ Subsequently, seven health states were defined which individuals could pass through or stay in during the model’s cycles (Table 1).

**Table 1.** Clinical outcome states according to the modified Rankin scale (mRS) with their respective assigned utility weights as applied in the Markov model^14,15^ (for this purpose, the mRS was collapsed into four categories).

mRS scale	State description	Utility weight	Collapsed state	Markov model state	Collapsed utility weight
0	No symptoms	0.95	Healthy	Healthy without aneurysm	0.93
				Healthy with aneurysm	0.93
				Healthy with aneurysm (f/u)	0.93
				Health with false neg. screening	0.93
1	No significant disability	0.90			0.93
2	Slight disability	0.83	Moderate disability	Moderate disability	0.76
3	Moderate disability	0.69			0.76
4	Moderate severe disability	0.38	Severe disability	Moderate disability	0.235
5	Severe disability	0.09			0.235
6	Dead	0	Dead	Moderate disability	0

f/u, aneurysm not requiring prophylactic treatment and therefore in follow-up; neg.: negative.

In addition, a database of patients with newly diagnosed UIAs who presented within a 2-year time frame was retrospectively collected (AC-UIA; 2022–2023; n = 139). The rupture risk of UIAs was calculated by means of the IAScore calculator and converted to a mean annual risk (Table 2).^10,16–19^ In case of aneurysm multiplicity, data from the most rupture-prone aneurysm were used.

**Table 2.** Patient- and aneurysm-specific data of all patients with incidentally found aneurysms presented in 2022 and 2023.

	All (n = 139)	Treated (n = 55)	Conservative (n = 84)	p
Demographics				
Age—yrs.—mean ± SD (range)	60.2 ± 13.4 (25-86)	58.2 ± 11.0 (29-85)	61.5 ± 14.7 (25-86)	0.160
Sex—Female / Male—no. (%)	99 (71.2) / 40 (28.8)	44 (80.0) / 11 (20.0)	55 (65.5) / 29 (34.5)	0.064
Aneurysm location—no. (%)				0.161
Acomm	12 (8.6)	4 (7.3)	8 (9.5)	
MCA	43 (30.9)	23 (41.8)	20 (23.8)	
ICA (incl. Pcomm)	61 (43.9)	18 (32.7)	43 (51.2)	
BA	6 (4.3)	3 (5.5)	3 (3.6)	
Others	17 (12.2)	7 (12.7)	10 (11.9)	
Post. circulation	11 (7.9)	6 (10.9)	5 (6.0)	0.290
Max. diameter (mm)—median (Q_1_ to Q_3_)	5.0 (3.0 to 7.0)	7.0 (5.0 to 9.0)	4.0 (3.0 to 6.0)	**<** **0.001**
Aneurysm occlusion—no. (%)				
Clipping / Endovascular	35 (25.2) / 18 (12.9)	35 (63.6) / 18 (32.7)	n/a	n/a
Both	2 (1.4)	2 (3.6)	n/a	
Aneurysm rupture risk—median (Q1 to Q3) (%)				
ISUIA annual	0 (0 to 0.520)	0.520 (0 to 0.520)	0 (0 to 0)	**<** **0.001**
UCAS annual	0.310 (0.140 to 1.190)	0.970 (0.310 to 1.560)	0.230 (0.140 to 0.310)	**<** **0.001**
PHASES annual	0.140 (0.140 to 0.260)	0.260 (0.140 to 0.480)	0.140 (0.140 to 0.180)	**<** **0.001**
Mean annual rupture risk	0.150 (0.093 to 0.630)	0.480 (0.150 to 0.807)	0.123 (0.075 to 0.260)	**<** **0.001**

Acomm, anterior communication artery; BA, basilar artery; ISUIA, International Study of Study of Unruptured Intracranial Aneurysms; mm, millimeters; PHASES, population hypertension age size of aneurysm earlier subarachnoid hemorrhage and site of aneurysm; Q_1_, first quartile; Q_2_, second quartile; SD, standard deviation; UCAS; unruptured cerebral aneurysms, yrs., years.

Significant p-values are written in bold.

In the combined AC-SAH and AC-UIA cohort of 414 patients, aneurysms were treated in 322 (77.8%) patients. Thereof, 143 patients (44.4%) received surgical clipping and 179 patients (55.6%) underwent endovascular occlusion. In the AC-SAH cohort, the average age of patients was 56.7 ± 12.9. With the tenth percentile at 41, screening initiated at the age of 40 years could identify 90% of patients prior to aneurysm rupture in this population. After SAH, 150 (54.5%) patients were rated as being in a state of favorable outcome after 1 year. Patient- and aneurysm-specific characteristics of both cohorts are summarized in Supplemental Table 2.

CEAs were performed in TreeAge Pro version 2024 R2.0 (TreeAge Software, Williamstown, MA, USA). We applied an annual discounting rate for costs and effects of 3.0%, based on recommendations for Germany.^20^ Individuals entered the model at the age of 40, accounting for 10% of potentially missed SAH in the PWS arm. Simulations progressed in 1-year cycles changing health states based on transition probabilities. During the model’s cycles, individuals might die of SAH or other unrelated causes. Age-related mortality was simulated for healthy, moderate-disabled, and severe-disabled individuals. Data were extracted from the national office for statistics (Statistisches Bundesamt) and imported into TreeAge Pro as a time-dependent variable (see Supplemental Appendix 1). Simulations were run until all individuals had passed away.

### PWS

In the PWS arm, a hypothetical diagnostic test is introduced as the initial screening modality. For baseline simulations, this test was assumed to be ideal, with perfect sensitivity and specificity (sensitivity = 1.0; specificity = 1.0). Comparative simulations were conducted to evaluate the performance of this hypothetical test against SOC approaches, as well as established imaging modalities—namely, magnetic resonance angiography (MRA; sensitivity = 0.85, specificity = 0.95) and computed tomography angiography (CTA; sensitivity = 0.90, specificity = 0.95).^21,22^ Two theoretical scenarios were modeled: one in which the diagnostic test required confirmatory imaging, and one in which it did not. In cases where a positive test result indicated the presence of an aneurysm (without assessing rupture risk), the cost of follow-up MRA was incorporated into the model. If MRA identified an aneurysm necessitating treatment based on rupture risk, additional diagnostic DSA costs were also included. The primary aim of this analysis was to estimate the cost of implementing the hypothetical diagnostic test relative to SOC. As such, PWS using CTA or MRA was not incorporated directly into the decision tree but was evaluated in separate pairwise comparisons. While the possibility of assessing net health/monetary benefits was considered (including CTA/MRA), it was ultimately excluded given the already established lack of cost-effectiveness for population-wide radiological screening. In this context, screening via imaging is mainly still included as a confirmatory tool of our model’s performance.^23^

### SOC

The SOC arm of the decision tree is based on the UKA treatment algorithms, where UIAs are followed yearly with MRA and every second year, if results remain stable for five consecutive years. Once treatment is indicated, patients undergo DSA to identify the best treatment strategy. Post-treatment, clipped patients receive DSA which is included in the initial hospital bill. From then on, patients are followed every 5 years with artifact-reducing CTA. Endovascularly treated patients receive MRA before discharge, and after 6, 18, and 36 months. Thereafter, follow-up is continued with MRA, every 3 years.

### Costs and probabilities

The UKA billing office provided final costs charged to the health insurance of all AC-SAH patients. This number included aneurysm and intensive care treatment as well as post-treatment imaging. Rehabilitation costs were estimated based on billing information provided by three main rehabilitation clinics within the UKA drainage area in relation to mRS at discharge. Annual costs of care for the disabled were estimated based on published monthly costs in Germany for either residential or non-residential care and adjusted for one year outcome (post-rehabilitation), age, and corrected by life expectancy.^24^ Costs of MRA and CTA were estimated based on billing information from neuroradiological services within the UKA catchment area. Total follow-up costs in the model are adjusted for outcome related average life expectancy since diagnosis of UIAs or SAH, and calculated separately for untreated, surgically or endovascularly treated UIAs, as well as SAH. Probabilities which could not be derived from both cohorts were based on literature reports in analogy with the works of Bor et al.^3^ and Hopmans et al.^4^ An overview of all costs and probabilities in the model, along with the source from which they are derived, is presented in Supplemental Table 3. Some accepted simplifying assumptions of the model are summarized in Supplemental Appendix 2.

### Standard protocol approval, registration, and patient consent

Prospective data collection was registered in the German Clinical Trial Register (DRKS00030505) and approved by the local ethics committee of the Medical Faculty of RWTH Aachen University (EK 14/062 and EK 22/371). Written informed consent was collected from all included patients or their legal representatives. Retrospective data acquisition was approved by our ethics committee (EK 23/317) and informed consent was waived due to the retrospective nature.

### Statistics and CEA analyses

Descriptive data are presented as mean and standard deviation (±SD) for normally distributed variables and as median with interquartile range (IQR: Q1 to Q3) for non-normally distributed variables. Nominal and ordinal data are reported as proportions (%). Normality was assessed using visual inspection and the Shapiro–Wilk test. Based on the distribution, appropriate statistical tests were applied: normally distributed continuous variables were compared using the unpaired t-test, while non-normally distributed variables were analyzed with the Mann–Whitney U-test. Nominal and ordinal variables were assessed using the χ^2^ test.

Statistical analyses were performed using IBM SPSS Statistics 29 (SPSS Inc., Chicago, IL, USA). Statistical significance was defined as a two-sided p < 0.05. Costs are reported in euros (€) to the cent, with a decimal point and commas as thousand separators. Incremental cost-effectiveness ratios (ICERs) were used to quantify the annual cost per QALY gained in the Markov model and to estimate the average cost per QALY for each arm.

ICER calculations were combined with a one-way sensitivity analysis to determine the price point at which the PWS strategy becomes either dominant (yielding a negative ICER) or cost-effective relative to three predefined willingness-to-pay (WTP) thresholds (€20,000, €50,000, or €100,000). The middle WTP threshold was based on recommendations from the World Health Organization, which considers a healthcare intervention cost-effective if the cost per QALY is below one times the country’s GDP per capita.^25^ As of 2022, Germany’s GDP per capita was €50,486.22, according to the World Bank.^26^ The upper WTP threshold was included for comparison, reflecting a commonly used benchmark in the United States.

## Results

### The decision tree

**Figure 1.**
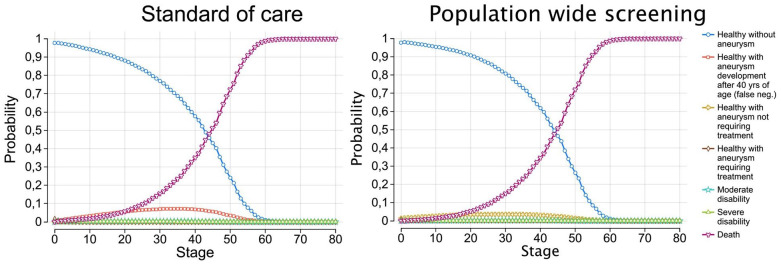
After 80 cycles, no patients were alive in neither the standard of care nor population-wide screening cohorts. neg., negative; yrs., years of age.

The full tree is included as Supplemental Figure 1. The simulated average life expectancy from age 40 was 41.3 years (95% confidence interval (CI) = 40.8–41.7), consistent with general population estimates. As shown in Figure 1, the probability of survival declines steadily, and all individuals had transitioned to the “Death” state by year 80, confirming a plausible mortality distribution without an overrepresentation of long-term survivors. In the SOC arm, costs and probabilities were held constant throughout the analysis. This arm includes costs associated with SAH treatment as well as follow-up costs for patients with incidental UIAs. The SOC strategy results in a total of 22.40 QALYs at an average cost of €3138.72 per patient.

### Performance of conventional screening techniques

In a scenario comparing SOC to PWS utilizing MRA once at age 40, the model yields an average cost of €23,013.24 for 22.52 QALYs gained (ICER = €169,383.57). When the same scenario is modeled using CTA—approximately €150 less per examination—the average cost decreases to €19,358.11 for 22.52 QALYs gained (ICER = €138,231.42). These findings serve as confirmation of the already well-established lack of cost-effectiveness associated with radiological PWS.

### Deterministic sensitivity analysis

**Figure 2.**
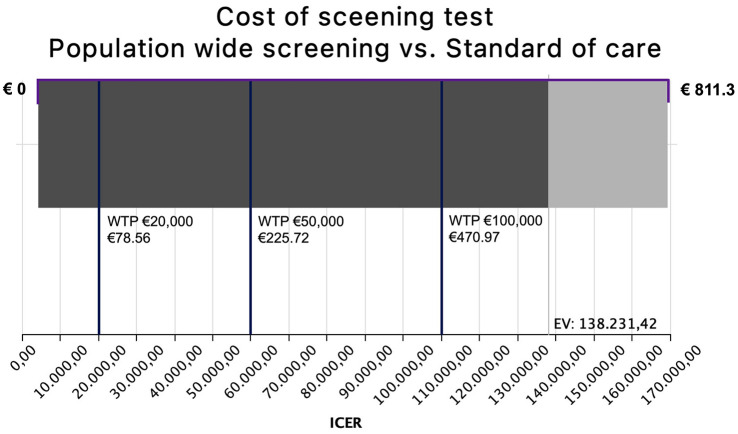
Tornado diagram depicting the range of ICERs achieved by varying the price of a hypothetical aneurysm screening test between €1 and €811.3 (mean cost of MRA) in a one-way sensitivity analysis. Here, a WTP of €20,000 per QALY gained can be achieved by a test costing €78.56, a WTP of €50,000 by a test costing €225.72, and a WTP of €100,000 by a test costing €470.97. There is no price point at which PWS would become the dominant strategy (negative ICER). The difference in grayscale of the bar chart indicates the expected value of the ICER’s probability distribution (€138,231.42). EV, expected value; ICER, incremental cost effects ratio; MRA, magnetic resonance angiography; PWS, population-wide screening; WTP, willingness to pay.

In a one-way sensitivity analysis, the cost of the hypothetical screening test was varied between €1 and €811.30 (the mean cost of MRA). Assuming a WTP threshold of €100,000 per QALY gained, the screening test would remain cost-effective at a maximum price of €470.97. At a WTP of €50,000, the test would need to cost no more than €225.72, while at a WTP of €20,000, the acceptable price drops to €78.56. There is no price point at which PWS becomes dominant, that is, yielding negative ICERs. These findings are visualized in a tornado diagram (Figure 2). Sensitivity and specificity ranges were not further explored, as deviations from a perfect test would only increase costs in the PWS arm, thereby further reducing its cost-effectiveness.

### Diagnostic test without the need for further confirmation

If a (blood based) test were to allow for rupture risk stratification and therefore reflect the necessity of treatment, this could bypass the cost of additional confirmational studies.^8^ A cost threshold analysis was run for a scenario in which the test would be used for initial screening as well as watchful-waiting in low-risk patients. In case of WTP €100,000 per QALY gained, this yields a cost threshold for the diagnostic test of €539.44. If the WTP were to decrease to €50,000, this threshold would come down to €294.19, and with a WTP of €20,000, it would decrease to €147.04. There is again no price point at which PWS would become dominant and yield negative ICERs.

### Populations at higher risk

In a second sensitivity analysis, the prevalence of UIAs at screening was varied between 2.3%^27^ and 9%,^28^ with corresponding increases in de novo aneurysm formation and rupture risk. Screening costs were capped at €811.30 (mean cost of MRA). At a prevalence of 4%—comparable to individuals with one first-degree relative—the ICER was €88,002.30. At a prevalence of 9%—as seen in those with two first-degree relatives—the ICER dropped to €18,746.30, falling below the lowest WTP threshold of €20,000 and thus rendering PWS cost-effective under these conditions.

### Probabilistic sensitivity analysis—Monte Carlo simulations

A Monte Carlo simulation was run, sampling 1000 iterations while applying underlying distribution parameters as reported in Supplemental Table 3. In a PWS scenario with a diagnostic test costing €225.72, this yields an expenditure of on average €10,308.99 per patient, for the generation of 22.46 QALYs. This is whereas the SOC scenario only costs €5,263.35, leading to 22.33 QALYs (ICER = 38,584.35). These results lie below a WTP €50,000 and prove comparable to our deterministic analysis (see above).

## Discussion

The cost-effectiveness of screening for UIA highly depends on both the cost of the screening tool as well as the a priori prevalence of aneurysms in the assessed population. In a sensitivity analysis, the price of a potential screening test was varied to identify at which price point, screening of the general population would become cost-effective. In the case of a WTP €50,000 per additional QALY, such a test may cost up to €225.72. If this test could also be used to continue watchful-waiting in low-risk patients, the price may increase up to €294.19 for the same WTP and remain cost-effective.

In this study, as much data as possible to input into the Markov model (i.e. costs and probabilities) were extracted from one real-world cohort. While sourcing input parameters from multiple heterogeneous populations is common practice and can increase the generalizability of model results, it may also introduce inconsistencies due to differences in baseline risk, clinical practices, and healthcare system structures. These inconsistencies can compromise the internal validity of the model, especially in conditions like UIAs, where epidemiological and cost data vary widely across countries. By relying on a homogeneous data set from a single, well-characterized population, we aimed to preserve consistency between epidemiological parameters and actual healthcare costs, thereby enhancing the internal coherence and interpretability of the model outcomes.

Four similar CEAs, using Markov models, have been published.^2–4,8^ In a study by Yoshimoto and Wakai,^2^ the cost of screening and treatment were based on rough estimates within the Japanese health care system. A sensitivity analysis was performed, not only varying the cost of treatment but also the prevalence of UIAs and incidence of aneurysm rupture. At an annual rupture rate of 0.01%, a screening strategy would be associated with a cost of $39,450 per QALY gained. In contrast, we considered these parameters as constants across the total population, which we believe better reflects clinical practice. This modeling decision was based on the fact that current rupture risk prediction tools do not incorporate age-specific rupture risk estimates. Instead, treatment decisions are typically based on aneurysm characteristics such as size and morphology. To date, no reliable epidemiological data exist that link these characteristics to rupture risk stratified by age. Two Dutch cost-effectiveness analyses focused on patients with a family history of SAH. In patients with two or more first-degree relatives who suffered SAH, screening from the age of 20 till 80 years old would prove cost-effective, given a WTP €20,000 per QALY gained.^3^ This is congruous to our results for individuals with high (9%) aneurysm prevalence. Hopmans et al.^4^ calculated that in patients with one first-degree relative who has suffered SAH, MRA is cost-effective when applied once at the age of 40 and once at the age of 55, given a WTP of €20,000.

In a CEA by Mittal et al.,^8^ the price of the diagnostic test for UIAs may cost up to $3,951 and remain cost-effective in case of WTP $34,515.13 per QALY gained, in a watchful-waiting population. Here, it may be argued that if the price of MRA undercuts that of the new diagnostic test, the latter remains obsolete. If, in our model, the WTP is set at this level, a price threshold of €200.44 (or $216,05) is generated. In this study, the hypothetical test is not only used as a screening tool but also to identify increasing rupture risk in a watchful-waiting subpopulation (with already confirmed UIAs). Herein, the authors assume that test result contains information not only on the presence but also on the rupture risk of UIAs. In addition, the model did not account for the fact that if treatment-worthy aneurysms are suspected by the test, confirmational radiological tests will remain necessary for treatment planning, which in turn generates costs.

### Limitations

Markov models and their associated cost-effectiveness analyses involve simplifications and have inherent methodological limitations. A key issue in our model is the estimation of utility values after SAH. Health outcomes were assessed using the mRS and converted to utility weights based on published mappings. However, the mRS primarily measures functional status and lacks the multidimensional perspective needed to fully capture health-related quality of life (HRQoL), especially emotional and social domains. While other instruments are better suited for this purpose, our outcome data—collected via structured telephone interviews—did not include a validated HRQoL measure. Consequently, we relied on literature-based utility values for mRS categories,^14^ which introduces a level of generalization. This may limit the precision of QALY estimates, particularly in reflecting psychological impacts. Future studies should incorporate validated HRQoL instruments to allow more comprehensive utility assessment.

Although we did not further explore test performance characteristics, future analyses may include two-way sensitivity analyses to define the combinations of sensitivity, specificity, and price under which a screening tool could become cost-effective, particularly if technological innovation lowers per-test costs. It is anticipated that imperfect real-world test sensitivity and specificity will result in higher price at which the test would be cost-effective.

In this model, all identified UIAs are systematically followed, generating costs. There are arguments in favor of not following up on small aneurysms with low estimated rupture risk. Both from a clinical and a public health perspective, the right follow-up protocol of UIAs is still controversially discussed.^29,30^ We opted to incorporate our institutional algorithm to further homogenize source data in the model.

As in most simulations, aneurysm prevalence and rupture risk for a given age were considered constant, and aneurysm growth, linear. There is epidemiological data underlining that above the age of 30, the prevalence of UIAs remains stable.^31^ Experimental and clinical research has pointed out that rupture risk may vary over time.^32,33^ The underlying age-related probability distributions are not precisely known and can therefore not be reliably implemented.

## Conclusion

If PWS were to be applied once at the age of 40, this strategy would prove cost-effective if a test is priced below €225.72 and there is a WTP €50,000 per QALY gained. If magnetic resonance imaging (MRI) were to be used for PWS, this would cost health care providers on average €169,383.57 per QALY gained. In subpopulations with a high a priori aneurysm prevalence, MRA screening is cost-effective even below WTP €20,000 per additional QALY.

These financial restrictions to render PWS for UIAs cost-effective can possibly not be overcome by newly developed technologies in the near future. However, once, for example, patents expire, and price monopolies are broken, lower prices may make reimbursement attractive for health policymakers, depending on their WTPs.

## Supplemental Material

sj-docx-1-wso-10.1177_17474930251344506 – Supplemental material for Cost-effectiveness of population-wide screening for intracranial aneurysms revisited in light of potential diagnostic developments Supplemental material, sj-docx-1-wso-10.1177_17474930251344506 for Cost-effectiveness of population-wide screening for intracranial aneurysms revisited in light of potential diagnostic developments by Michael Veldeman, Oliver Schoeffski, Anke Hoellig and Gabriel J E Rinkel in International Journal of Stroke

sj-pdf-2-wso-10.1177_17474930251344506 – Supplemental material for Cost-effectiveness of population-wide screening for intracranial aneurysms revisited in light of potential diagnostic developments Supplemental material, sj-pdf-2-wso-10.1177_17474930251344506 for Cost-effectiveness of population-wide screening for intracranial aneurysms revisited in light of potential diagnostic developments by Michael Veldeman, Oliver Schoeffski, Anke Hoellig and Gabriel J E Rinkel in International Journal of Stroke
