# Supplementary material for: Cost-effectiveness of population-wide screening for intracranial aneurysms revisited in light of potential diagnostic developments
Source: Int J Stroke. 2025 May 12;20(9):1132–40. doi: 10.1177/17474930251344506 (PMC12521774; doi:10.1177/17474930251344506)
Supplement: sj-docx-1-wso-10.1177_17474930251344506 – Supplemental material for Cost-effectiveness of population-wide screening for intracranial aneurysms revisited in light of potential diagnostic developments [file sj-docx-1-wso-10.1177_17474930251344506.docx]

**Supplemental Material**

**Supplemental Table 1**

| **Municipality** | **Inhabitants as of 2022** | **Surface area in km²** |
| --- | --- | --- |
| Aachen, Stadt | 252,136 | 161 |
| Aldenhoven | 14,081 | 44.3 |
| Alsdorf, Stadt | 48,328 | 31.7 |
| Baesweiler, Stadt | 27,620 | 27.8 |
| Düren, Stadt | 93,207 | 85 |
| Erkelenz, Stadt | 44,215 | 117 |
| Eschweiler, Stadt | 56,049 | 75.8 |
| Gangelt | 13,240 | 48.7 |
| Geilenkirchen, Stadt | 28,252 | 83.2 |
| Heimbach, Stadt | 4,365 | 65 |
| Heinsberg, Stadt | 43,476 | 92.2 |
| Herzogenrath, Stadt | 46,941 | 33.4 |
| Hückelhoven, Stadt | 41,301 | 61.3 |
| Hürtgenwald | ,8929 | 88.1 |
| Inden | 7,507 | 35.9 |
| Jülich, Stadt | 33,158 | 90.4 |
| Kreuzau | 17,687 | 41.7 |
| Langerwehe | 14,257 | 41.5 |
| Linnich, Stadt | 13,015 | 65.4 |
| Merzenich | 10,302 | 37.9 |
| Monschau, Stadt | 11,864 | 94.6 |
| Nideggen, Stadt | 10,419 | 65 |
| Niederzier | 14,345 | 63.5 |
| Nörvenich | 11,064 | 66.2 |
| Roetgen, Tor zur Eifel | 8,727 | 39 |
| Selfkant | 10,557 | 42.1 |
| Simmerath | 15,841 | 111 |
| Stolberg (Rhld.), Kupferstadt | 56,455 | 98.5 |
| Titz | 8,686 | 68.6 |
| Übach-Palenberg, Stadt | 2,4215 | 26.1 |
| Vettweiß | 9,811 | 83.2 |
| Waldfeucht | 9,164 | 30.3 |
| Wassenberg, Stadt | 1,9339 | 42.4 |
| Wegberg, Stadt | 28,074 | 84.3 |
| Würselen, Stadt | 38,598 | 34.4 |
| **Total** | 1,095,225 | 2,276 |

**Supplemental Table 1.** Municipalities of the catchment area for neurosurgical services in the treatment of aneurysmal subarachnoid hemorrhage, provided by the RWTH University Hospital Aachen, Germany (in alphabetical order). Data is collected by the National Institute for Construction- City and Spatial Planning (Bundesinstitut für Bau-, Stadt- und Raumforschung (BBSR) (1).

km^2^, square kilometers.

**Supplemental Table 2.**

|  | **All patients (n = 414)** | **UIA (n = 139)** | **SAH (n = 275)** | **p-value** |
| --- | --- | --- | --- | --- |
| **Demographics** |  |  |  |  |
| Age - yrs. - mean ± SD (range) | 57.8 ± 13.1 (19- 90) | 60.2 ± 13.4 (25- 86) | 56.7 ± 12.9 (19- 90) | **0.001** |
| Sex - Female / Male - no. (%) | 280 (67.6) / 134 (32.4) | 99 (71.2) / 40 (28.8) | 181 (65.8) / 94 (34.2) | 0.267 |
| **Aneurysm location - no. (%)** |  |  |  | **< 0.001** |
| Acomm | 97 (23.4) | 12 (8.6) | 85 (30.9) |  |
| MCA | 112 (27.1) | 43 (30.9) | 69 (25.1) |  |
| ICA (incl. Pcomm) | 125 (30.2) | 61 (43.9) | 64 (23.3) |  |
| BA | 23 (5.6) | 6 (4.3) | 17 (6.2) |  |
| Others | 57 (13.8) | 17 (12.2) | 40 (14.5) |  |
| Post. circulation | 219 (13.8) | 11 (7.9) | 60 (21.8) | 0.001 |
| Max. diameter (mm) - median (Q_1_ - Q_3_) | 6.0 (4.0 - 8.0) | 5.0 (3.0 to 7.0) | 6.0 (4.5 - 9.0) | **<0.001** |
| **Aneurysm occlusion - no. (%)** |  |  |  |  |
| Clipping / Endovascular | 143 (34.5) / 179 (43.2) | 35 (25.2) / 18 (12.9) | 108 (39.3) / 161 (58.5) | **<0.001** |
| Both | 5 (1.2) | 2 (1.4) | 3 (1.1) |  |
| No occlusion | 87 (21.0) | 84 (60.4) | 3 (1.1) |  |
| **Hemorrhage severity** |  |  |  |  |
| Poor-grade SAH (WFNS 3-5) | n/a | n/a | 100 (36.4) |  |
| **Clinical outcome** |  |  |  |  |
| **mRS 12 months - no. (%)** |  |  |  |  |
| Death | n/a | n/a | 63 (22.9) |  |
| Severe disability | n/a | n/a | 12 (4.4) |  |
| Moderate severe disability | n/a | n/a | 22 (8.0) |  |
| Moderate disability | n/a | n/a | 17 (6.2) |  |
| Slight disability | n/a | n/a | 31 (11.3) |  |
| No disability | n/a | n/a | 45 (16.4) |  |
| No symptoms | n/a | n/a | 57 (20.7) |  |
| Favorable outcome (mRS 0-3) | n/a | n/a | 150 (54.5) |  |
| Missing | n/a | n/a | 28 (10.2) |  |

**Supplemental Table 2.** Direct comparison of patients in the UIA and SAH cohorts.

Acomm, anterior communication artery; BA, basilar artery; ICA, internal carotid artery; MCA, middle cerebral artery; mm, millimeters; mRS, modified rankin scale; Pcomm, posterior communicating artery; Q_1_, first quartile; Q_3_, third quartile; SAH, aneurysmal subarachnoid hemorrhage; SD, standard deviation; UIA, unruptured intracranial aneurysm; WFNS, World Federation of Neurosurgical Societies.

**Supplemental Table 3**

| **Variable** | **Source** | **Cohort size** | **Probability / Mean cost (euro)** | **Type of distribution** | **Standard Deviation** |
| --- | --- | --- | --- | --- | --- |
| **State and transition probabilities (per cycle / year if applicable)** |  |  |  |  |  |
| Aneurysm incidence | Literature |  | 0.023 | binomial |  |
| Aneurysm de novo formation | Literature |  | 0.003 | binomial |  |
| Aneurysm rupture risk for aneurysm not requiring treatment | Literature |  | 0.005 | binomial |  |
| Aneurysm found incidentally | AC-UIA & AC-SAH | Hospitals drainage area | 0.00006 | binomial |  |
| Aneurysm growth in incidental UIA | Literature |  | 0.025 | binomial |  |
| Aneurysm rupture risk in aneurysm requiring treatment | Literature |  | 0.480 | binomial |  |
| Requiring endovascular aneurysm treatment | AC-UIA & AC-SAH | 330 | 0.556 | binomial |  |
| Requiring surgical aneurysm treatment | AC-UIA & AC-SAH | 330 | 0.444 | binomial |  |
| Mortality SAH | AC-SAH | 275 | 0.255 | age related |  |
| Severe disability after SAH | AC-SAH | 275 | 0.138 | binomial |  |
| moderate disability after SAH | AC-SAH | 275 | 0.194 | binomial |  |
| Healthy after SAH | AC-SAH | 275 | 0.413 | binomial |  |
| Mortality of severe disability | Literature | n/a |  | age related |  |
| Mortality of moderate disability | Literature | n/a |  | age related |  |
| Mortality healthy | Bundesamt für Statistik, NRW | n/a |  | age related |  |
| Moderate disability preventive clipping | AC-UIA | 35 | 0.029 |  |  |
| Severe disability preventive clipping | AC-UIA | 35 | 0.029 | binomial |  |
| Mortality preventive clipping | AC-UIA | 35 | 0.029 | binomial |  |
| Moderate disability preventive endovascular treatment | AC-UIA | 18 | 0.056 | binomial |  |
| Severe disability preventive endovascular treatment | AC-UIA | 18 | 0.001 | binomial |  |
| Mortality preventive endovascular treatment | AC-UIA | 18 | 0.001 | binomial |  |
| **Costs** |  |  |  |  |  |
| Cost CTA | Questionnaire |  | 658.50 | Gamma | 211.28 |
| Cost MRA | Questionnaire |  | 811.30 | Gamma | 161.18 |
| Cost confirmational DSA | AC-UIA |  | 2112.34 | Gamma | 134.67 |
| Annual cost care for patient with severe disability | Literature | n/a | 37,1 |  |  |
| Annual cost care for patient with moderate disability | Literature | n/a | 5424 |  |  |
| Cost preventive endovascular aneurysm treatment | AC-UIA | 18 | 17,808.94 | Gamma | 16,983.06 |
| Cost preventive surgical aneurysm treatment | AC-UIA | 35 | 18,447.95 | Gamma | 13,199.08 |
| Cost SAH total | AC-SAH | 275 | 154,978.92 | Gamma | 228,813.52 |
| **Utilities** |  |  |  |  |  |
| Utility of being healthy | Literature | n/a | 0.94 | Beta | 0.05 |
| Utility of severe disability | Literature | n/a | 0.265 | Beta | 0.05 |
| Utility of moderate disability | Literature | n/a | 0.725 | Beta | 0.05 |
| Utility of death | Literature | n/a | 0 |  |  |

**Supplemental Table 3.** Overview of input data for the Markov model and its sources.

AC-SAH, prospective Aachen cohort of SAH patients; AC-UIA, retrospective Aachen cohort of UIA patients; CTA, computed tomography angiography; NRW, North Rhine-Westphalia; MRA, magnetic resonance angiography; n/a, not applicable; SAH, aneurysmal subarachnoid hemorrhage.

**Appendix 1.**

**Mortality in the disabled**

The estimation of mortality figures in patients with a disability after suffering either SAH or an UIA treatment complication, is more complex. Available data commonly refer to populations of intellectually disabled individuals and not specifically to people with an acquired intellectual of physical disability related to brain injury (2). On average the life expectancy is estimated to be 13 years shorter compared to the general population. To generate age specific mortality figures, the table of age-related mortality in the German population was shifted 13 years for patients with severe disability and 5 years for people with moderate disability.

**Appendix 2.**

**Accepted simplifying assumptions**

Between 2014 and 2020, Germany has known an average inflation rate of 1.03%. This rate was used to correct all cost for inflation on a yearly basis bringing all cost up to a 2024 level (3). Apart from discounting within the Markov model, future costs were not corrected for inflation as all cost are assess as of 2024. All costs are expressed in euro (€) to the cent using a decimal point and commas as thousand separators.

In the initial exploratory analyses, no differences in state and transition probabilities between sexes were accounted for and therefore not implemented in the final Markov model.

We assume that once an UIA develops, it will not heal or disappear by itself. The prevalence of UIAs as found in screening examinations as well as the prevalence of incidental UIA findings will be considered stable for all ages. The likelihood of developing are carrying an UIA increases with age (4). Nonetheless, the estimation of an age variable prevalence is cumbersome due to limited exiting data on *de novo* aneurysm formation in the general population. Most aneurysms are diagnosed in people between the ages of 35 and 60 years (5). A peak of incidental UIAs findings is observed in individuals between the ages of 50 and 60 (6). Acknowledging this simplification, the risk of carrying UIAs will be kept constant, independent of age.

The possibility of false negative or false positive findings during aneurysm screening, will not be taken into account, as the screening test itself is purely hypothetical.

Many indirect and hidden costs of disability are difficult to estimate and are not accounted for (7). This includes (not exhaustive): community and societal costs due to loss of productive years, decrease in levels of earnings due to disability or limited access to gain and retain employment, opportunity costs of foregone income for family members giving up work to provide support etc..

1. Bundesinstitut für Bau-, Stadt- und Raumforschung [Available from: <https://www.bbsr.bund.de/BBSR/DE/startseite/_node.html>.

2. Heslop P, Blair PS, Fleming P, Hoghton M, Marriott A, Russ L. The Confidential Inquiry into premature deaths of people with intellectual disabilities in the UK: a population-based study. Lancet. 2014;383(9920):889-95.

3. German inflation data [Available from: <https://www.globaldata.com/data-insights/macroeconomic/inflation-rate-in-germany/#:~:text=Between%202010%2D2021%2C%20the%20consumer,oil%20and%20motor%20fuel%20prices>.

4. Brinjikji W, Zhu YQ, Lanzino G, Cloft HJ, Murad MH, Wang Z, et al. Risk Factors for Growth of Intracranial Aneurysms: A Systematic Review and Meta-Analysis. AJNR Am J Neuroradiol. 2016;37(4):615-20.

5. Juvela S, Porras M, Poussa K. Natural history of unruptured intracranial aneurysms: probability of and risk factors for aneurysm rupture. J Neurosurg. 2000;93(3):379-87.

6. Wong CKH, O'Rielly CM, Sheppard B, Beller G. The emergency department incidence of incidental intracranial aneurysm on computed tomography angiography (EPIC-ACT) study. Cjem. 2022;24(3):268-72.

7. Soltani S. Hidden cost of disability: What policy makers usually forget. J Res Med Sci. 2018;23:15.
