# Supplementary material for: Cost-effectiveness of population-wide screening for intracranial aneurysms revisited in light of potential diagnostic developments
Source: Int J Stroke. 2025 May 12;20(9):1132–40. doi: 10.1177/17474930251344506 (PMC12521774; doi:10.1177/17474930251344506)
Supplement: sj-pdf-2-wso-10.1177_17474930251344506 – Supplemental material for Cost-effectiveness of population-wide screening for intracranial aneurysms revisited in light of potential diagnostic developments [file sj-pdf-2-wso-10.1177_17474930251344506.pdf]

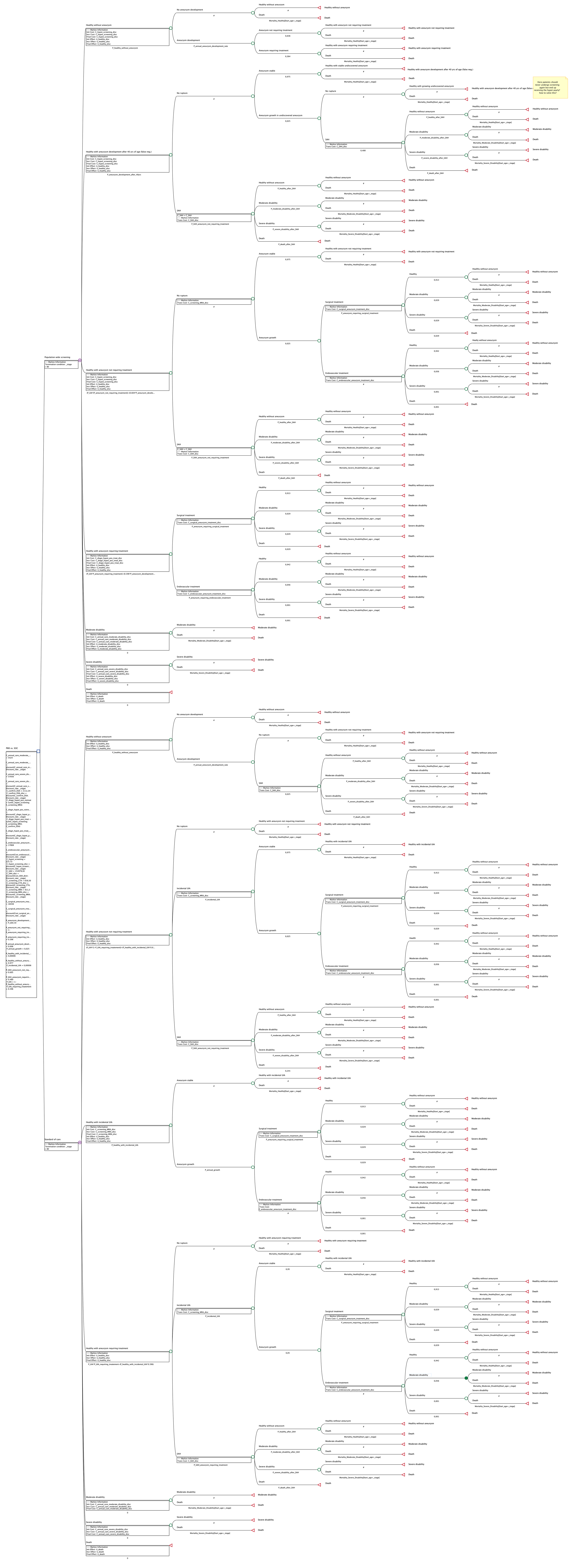
PWS vs. SOC
C_annual_care_moderate_... = 5424
C_annual_care_moderate_... = discount(C_annual_care_m... Discount_rate; _stage)
C_annual_care_severe_dis... = 37056
C_annual_care_severe_dis... = discount(C_annual_care_s... Discount_rate; _stage)
C_confirm_DSA = 2112,34
C_confirm_DSA_disc = discount(C_confirm_DSA; Discount_rate; _stage)
C_diagn_hypot_pos_notreat = sum(C_hypot_screening; C_screening_MRA)
C_diagn_hypot_pos_notre... = discount(C_diagn_hypot_p... Discount_rate; _stage)
C_diagn_hypot_pos_treat = sum(C_hypot_screening; C_screening_MRA; C_confirm_DSA)
C_diagn_hypot_pos_treat_... = discount(C_diagn_hypot_p... Discount_rate; _stage)
C_endovascular_aneurysm... = 17808
C_endovascular_aneurysm... = discount(Cost_endovascul... Discount_rate; _stage)
C_hypot_screening = 225.72
C_hypot_screening_disc = discount(C_hypot_screeni... Discount_rate; _stage)
C_SAH = 154978,92
C_SAH_disc = discount(Cost_SAH_distr; Discount_rate; _stage)
C_screening_CTA = 658,50
C_screening_CTA_disc = discount(C_screening_CTA; Discount_rate; _stage)
C_screening_MRA = 811,3
C_screening_MRA_disc = discount(C_screening_MRA; Discount_rate; _stage)
C_surgical_aneurysm_trea... = 18448
C_surgical_aneurysm_trea... = discount(Cost_surgical_an... Discount_rate; _stage)
P_aneurysm_development... = P_UIA/10
P_aneurysm_not_requiring... = 1-P_aneurysm_requiring_tre...
P_aneurysm_requiring_tre... = 0.396
P_annual_aneurysm_devel... = 0.006
P_annual_growth = 0,025
P_healthy_with_incidental_... = 0,00006
P_healthy_without_aneury... = 0.977
P_incidental_UIA = 0,00006
P_SAH_aneurysm_not_req... = 0.005
P_SAH_aneurysm_requirin... = 0.480
P_UIA = 1-P_healthy_without_aneury...
P_UIA_requiring_treatement = 0.396
Population wide screening
--- Markov Information
Termination condition: _stage = 80
Standard of care
--- Markov Information
Termination condition: _stage = 80
Here pateints should never undergo screening again but end up receiving the hypot yearly? how to solve this?
